# Factors associated with human papilloma virus vaccine uptake before the health awareness, learning and education intervention in Korogocho Informal Settlements in Nairobi County, Kenya

**DOI:** 10.3332/ecancer.2025.1892

**Published:** 2025-04-15

**Authors:** Jared Otieno Ogolla, David Rocaztle Masinde, Alexander Munyao Mbeke

**Affiliations:** 1 Department of Public Health, School of Public Health and Community Development, Maseno University, Private Bag, Maseno, Kenya; 2 Department of Biological and Biomedical Sciences, School of Science, Laikipia University, PO Box 1100-20300, Nyahururu, Kenya

**Keywords:** human papillomavirus, HPV vaccine, HPV vaccine uptake, HEALEDUC

## Abstract

The human papillomavirus (HPV) vaccine is a critical public health measure designed to reduce the incidence of HPV-related cancers, particularly cervical cancer. Despite its proven efficacy, vaccine uptake remains suboptimal in many areas, including Korogocho ward, a densely populated informal settlement in Nairobi County, Kenya. This study explored factors influencing HPV vaccine uptake among girls aged 9–14 in Korogocho. A total of 812 caretakers participated, identified through snowball sampling during the baseline survey of the Health Awareness, Learning and Education (HEALEDUC) intervention. The HEALEDUC initiative, a quasi-experimental study, employed intervention and control groups with pre- and post-intervention assessments to evaluate strategies for improving HPV vaccination rates in the region. Key findings revealed that caretaker age significantly impacted vaccination decisions (*p* = 0.022). Caretakers aged 35–44 were more likely to vaccinate their children (OR = 1.930, 95% CI = 0.790–4.716), although no consistent patterns emerged among other age groups. Interestingly, uncertainty about HPV transmission was associated with higher vaccine uptake (OR = 2.024, 95% CI = 1.107–3.701, *p* = 0.022). Negative perceptions of healthcare workers’ attitudes strongly correlated with increased vaccination likelihood (OR = 4.883, 95% CI = 1.834–12.999, *p* = 0.002). Satisfaction with healthcare services demonstrated borderline significance (*p* = 0.059). Conversely, distance to healthcare facilities (*p* = 0.348) and transport costs (*p* = 0.873) were not statistically significant determinants of vaccine uptake. However, caretakers residing more than 10 km from healthcare facilities exhibited slightly higher odds of vaccinating their children (OR = 3.136, 95% CI = 0.521–18.881). These findings underscore the importance of targeted interventions to bridge knowledge gaps, foster trust in healthcare systems and improve interactions between caretakers and healthcare providers. Addressing these factors can enhance HPV vaccine uptake in resource-limited settings.

## Background

The human papillomavirus (HPV) vaccine is a critical public health intervention for reducing HPV-related cancers, particularly cervical cancer [1, 2]. Despite its efficacy, its uptake remains low in regions like Korogocho ward, a densely populated informal settlement in Nairobi County, Kenya [3]. This gap in vaccine coverage presents significant public health challenges, particularly in areas with heightened vulnerability to HPV-related health risks.

Korogocho, situated in Ruaraka Sub-County, is characterised by severe socio-economic hardships, including overcrowding, insufficient infrastructure and limited access to healthcare services [3–5]. These factors contribute to poor health outcomes and low vaccination rates [5, 6]. Poverty, unemployment and underemployment further restrict access to essential services like the HPV vaccine, perpetuating health inequities [3].

HPV-related risks in Korogocho are acute. Early sexual debut and high rates of sexual violence increase girls’ vulnerability to HPV infection [5–7]. By 2015, only 30% of girls aged 9–14 in Korogocho had received the HPV vaccine, well below the national average [3]. Addressing barriers to uptake requires understanding individual, sociocultural and systemic challenges.

Knowledge gaps, beliefs and perceived risks significantly influence vaccine uptake. Studies in sub-Saharan Africa show that individuals aware of HPV and its vaccine are more likely to seek vaccination [8]. In Kenya, limited awareness remains a major barrier [8–10].

Sociocultural factors, including community norms, religious beliefs and vaccine misconceptions, also hinder uptake [11–14]. In low-resource settings, cultural stigmas and misinformation further undermine vaccination efforts [12].

Systemic challenges, such as weak vaccine supply chains, inadequate cold storage and healthcare worker shortages, hinder delivery [15, 16]. These issues disproportionately affect marginalised areas like Korogocho, where healthcare access is already limited.

This study investigated factors influencing HPV vaccine uptake among girls aged 9–14 in Korogocho to identify barriers and inform targeted interventions to improve vaccination coverage and health outcomes.

## Methods

### Study area

Korogocho faces significant socio-economic and healthcare challenges [3, 17], including overcrowding, poverty and limited access to healthcare, which contribute to the high prevalence of sexually transmitted infections such as HPV [3, 5–7]. Early sexual debut, high school dropout rates and cultural beliefs further exacerbate these issues [6, 7], underscoring the urgent need for targeted health education and vaccination interventions.

### Study design

Data on barriers to HPV vaccine uptake were obtained from the baseline survey of the Health Awareness, Learning and Education intervention, a quasi-experimental study in which intervention and control groups were assessed pre-intervention and post-intervention to evaluate strategies for improving HPV vaccination rates. Currently, the HPV vaccines are provided at no cost to girls below the age of 14 years in all public facilities in the country.

### Target population and sample size

The study targeted caretakers of girls aged 9–14, with 812 participants identified through snowball sampling.

### Data collection tools

Data were collected using an interviewer-administered questionnaire that captured the caretakers’ socio-demographic characteristics, including age, education, occupation, income and housing. The questionnaire also assessed their knowledge of HPV and the vaccine, including transmission, benefits and availability.

Attitudes toward vaccination were explored, focusing on perceived costs, side effects and responsibility. Social, cultural and health system factors were also investigated, including decision-making dynamics, cultural beliefs, health worker attitudes and logistical barriers such as transport and distance.

### Data analysis

Bivariate analysis was used to analyse factors associated with HPV vaccine uptake. Factors that showed significant associations (*p* < 0.05) were then included in a binary logistic regression model to control for confounding variables and assess the predictive strength of these factors.

## Results

### Characteristics of the study participants

Most caretakers were young, with 11.8% aged 18–25 and the largest group (33.8%) in the 26–34 range. Children’s ages were almost evenly split, with 50.9% aged 9–11 and 49.1% aged 12–14. Over half of caretakers (53.3%) had no formal schooling, and only 1.0% pursued tertiary education. More than half (50.6%) worked informally, and 71.1% earned KSh. ≤ 20,000. In housing, 47.6% lived in semi-permanent structures and 27.6% resided in mud or iron sheet-walled houses. These findings are summarised in Table 1.

### Sociodemographic factors associated with HPV vaccine uptake

The caretakers’ age was significantly associated with HPV vaccine uptake (*χ*²(4, *N* = 812) = 10.927, *p* = 0.027). However, other factors, including the child’s age (*χ*²(1, *N* = 812) = 1.515, *p* = 0.218), the caretaker’s educational level (*χ*²(3, *N* = 812) = 1.590, *p* = 0.662), occupation (χ²(2, *N* = 812) = 3.492, *p* = 0.174), monthly income (*χ*²(5, *N* = 812) = 6.370, *p* = 0.272) and housing type (*χ*²(2, *N* = 812) = 2.031, *p* = 0.362), were not significantly associated with vaccine uptake. See Table 2.

### Socio-cultural factors as a predictor of HPV vaccine uptake

Several factors did not significantly influence vaccination uptake. These included seeking permission for vaccination (*χ*²(2, *N* = 812) = 1.600, *p* = 0.449), the individual the caretaker consulted before vaccinating the child (*χ*²(7, *N* = 812) = 5.190, *p* = 0.637), the presence or absence of a spouse during vaccination sessions (*χ*²(2, *N* = 812) = 0.095, *p* = 0.953), reasons for a spouse’s absence (χ²(5, *N* = 812) = 2.348, *p* = 0.799), cultural beliefs opposing vaccination (*χ*²(2, *N* = 812) = 0.457, *p* = 0.796) and concerns about vaccines such as fears of infertility or beliefs that vaccines might encourage early sexual activity (*χ*²(5, *N* = 812) = 4.489, *p* = 0.481). Refer to Table 3.

### Knowledge of the caretakers as a predictor of HPV vaccine uptake

Caretakers’ understanding of HPV transmission through anal or vaginal intercourse was significantly associated with their decision to vaccinate their children (*χ*²(2, *N* = 812) = 10.937, *p* = 0.004). However, awareness of warts as a sign of HPV infection (*χ*²(2, *N* = 812) = 4.742, *p* = 0.093), knowledge of other symptoms such as vaginal bleeding during or after intercourse (*χ*²(2, *N* = 812) = 0.998, *p* = 0.607) and awareness of causes like having multiple sexual partners or a weakened immune system (*χ*²(2, *N* = 812) = 4.282, *p* = 0.118) did not significantly influence vaccination uptake.

Additionally, caretakers’ understanding of the HPV vaccine’s role in preventing cervical cancer (*χ*²(2, *N* = 812) = 0.103, *p* = 0.950), the necessity of Pap tests as part of HPV prevention (*χ*²(2, *N* = 812) = 1.237, *p* = 0.539), knowledge of the recommended age for HPV vaccination (*χ*²(2, *N* = 812) = 0.941, *p* = 0.625), the availability of free HPV vaccines at public health facilities (*χ*²(2, *N* = 812) = 0.973, *p* = 0.615) and the readiness of public health facilities to provide the vaccine (*χ*²(2, *N* = 812) = 2.204, *p* = 0.332) did not significantly impact caretakers’ decisions. See Table 4.

### Attitude of the caretakers as a predictor of HPV vaccine uptake

Caretakers’ belief that the vaccine was expensive (*χ*²(4, *N* = 812) = 5.160, *p* = 0.271), their awareness of its ability to prevent cervical cancer (*χ*²(4, *N* = 812) = 4.552, *p* = 0.336) and misconceptions about the vaccine causing infertility (χ²(4, *N* = 812) = 1.982, *p* = 0.739) were not significant determinants. Similarly, caretakers fears about the vaccine causing serious infections (*χ*²(4, *N* = 812) = 5.150, *p* = 0.272), their perceptions of the appropriate age for vaccination (*χ*²(4, *N* = 812) = .663, *p* = 0.956), concerns about pain during administration (*χ*²(4, *N* = 812) = 2.408, *p* = 0.661) and a sense of responsibility to ensure their children were vaccinated (*χ*²(4, *N* = 812) = 2.638, *p* = 0.620) showed no significant association with vaccination decisions. These findings have been summarised in Table 5.

### Health facility factors as a predictor of HPV vaccine uptake

Among the 812 caretakers surveyed during the pretest, 448 reported having taken a child for vaccination at some point. However, prior vaccination experience (*χ*²(1, *N* = 812) = 0.397, *p* = 0.529), the time elapsed before a child received prior vaccinations (*χ*²(3, *N* = 448) = 2.003, *p* = 0.572) and missing any vaccine at the facility (*χ*²(2, *N* = 448) = 1.803, *p* = 0.406) did not significantly influence caretakers’ vaccination decisions. Logistical challenges, including missed opportunities due to vaccine shortages, absent health workers, overcrowded facilities or long waiting times, were also not significantly associated with HPV vaccination uptake during the pretest (*χ*²(3, *N* = 109) = 1.173, *p* = 0.759).

Conversely, caretakers’ perceptions of health workers (*χ*²(3, *N* = 448) = 8.399, *p* = 0.038), satisfaction with health facility services (*χ*²(2, *N* = 448) = 7.319, *p* = 0.026) and accessibility factors, such as the distance to health facilities (*χ*²(3, *N* = 448) = 19.485, *p* < 0.001) and transportation costs (*χ*²(3, *N* = 448) = 17.609, *p* < 0.001), were significantly associated with vaccination outcomes. See Table 6.

### Regression analysis

Regression analysis revealed that caretakers aged 26–34 were slightly less likely to vaccinate their children against HPV compared to those aged 18–25 (OR = 0.821, 95% CI = 0.340–1.981, *p* = 0.660), while those aged 35–44 were somewhat more likely (OR = 1.930, 95% CI = 0.790–4.716, *p* = 0.149). The same lack of a clear pattern emerged among those aged 45–54 (OR = 1.461, 95% CI = 0.568–3.760, *p* = 0.431) and ≥ 55 (OR = 1.924, 95% CI = 0.407–9.094, *p* = .409). Such results suggest that age, in isolation, does not appear to shape HPV vaccination decisions consistently. One participant noted,

*‘It seems that younger parents, particularly my age group, often hesitate more because they feel uncertain. They also lack the resources or knowledge to make an informed decision about vaccinations. However, older caretakers may have more exposure to the consequences of diseases like cervical cancer, which strengthens their resolve. They also tend to be more decisive about their children’s health.’ (FGD 1, Female 23 years)*

Notwithstanding, caretakers who were uncertain if HPV could be transmitted via anal or vaginal route were significantly more likely to vaccinate their children (OR = 2.024, 95% CI = 1.107–3.701, *p* = 0.022). In contrast, those who negated this statement showed no significant difference in vaccination behaviour (OR = 1.132, 95% CI = 0.690–1.858, *p* = 0.623). One caretaker lamented,

*‘Sometimes, lacking all the facts makes you more cautious, and that caution drives you to take action to protect your child. It is better to be safe than sorry. It is just good to avoid regrets in future for not acting.’ (FGD 2, Male 38 years)*

From this finding, it appears that uncertainty about HPV transmission might have encouraged a more cautious approach, prompting caretakers to opt to vaccinate their daughters as a preventive measure. Another parent shared,

*‘I didn’t understand all the specifics of HPV transmission, but when the CHVs explained that the vaccine could prevent cervical cancer, I felt it was enough for me to get my daughter vaccinated. When in doubt, I’d rather take the safer route, and the vaccine sounded like the safest option.’ (FGD 3, Female 41 years)*

These reflections highlight that limited knowledge about HPV transmission can sometimes propel caretakers to prioritise vaccination as a precaution. The study also observed that caretakers who rated the attitude of health workers as ‘Poor’ were more likely to vaccinate their children (OR = 4.883, 95% CI = 1.834–12.999, *p* = 0.002) compared to those who rated it as ‘Good.’ One caretaker remarked,

*‘The nurses were cold and unhelpful, but I ensured my child got vaccinated. I couldn’t allow a negative interaction with a health worker to prevent me from doing what I thought was best for my child’s future.’ (FGD 4, Female 35 years)*

This counterintuitive finding suggests that dissatisfaction with health-worker interactions can sometimes drive caretakers to take proactive measures. Another participant explained,

*‘The lack of kindness from health workers sometimes motivates me to get the vaccination done as quickly as possible. I didn’t want to go back to that facility, so I made sure I finished what I started with my child’s vaccine.’ (FGD 5, Female 44 years)*

These experiences reveal that health worker attitudes can influence caretakers’ HVP vaccination decisions in ways that may not be clear. Caretakers who rated the attitude of health workers as ‘Average’ showed moderately higher odds of vaccination (OR = 1.704, 95% CI = 0.901–3.223, *p* = 0.101), though this was not statistically significant. Interestingly, caretakers who were unsure about the health workers’ attitudes were less likely to vaccinate (OR = 0.725, 95% CI = 0.349–1.507, *p* = 0.389), signalling that clarity and trust in health workers are vital for influencing vaccination choices.

Interestingly, satisfaction with healthcare services had a weaker, yet noticeable, influence on vaccination behaviour. Caretakers dissatisfied with services showed no significant difference in vaccination behaviour compared to those satisfied (OR = 0.969, 95% CI = 0.497–1.890, *p* = 0.926). However, those who were uncertain about their level of satisfaction (‘Don’t know’) were more likely to vaccinate their children, with borderline statistical significance (OR = 2.476, 95% CI = 0.967–6.342, *p* = 0.059). One caretaker shared,

*‘I was a little bit unhappy with the services, but when it comes to my child’s health, I couldn’t wait around for things to improve. I didn’t feel it was a reason to delay the vaccination.’ (FGD 6, Male 29 years)*

Ambiguity regarding satisfaction does not deter caretakers from vaccinating. Rather, it may increase the urgency to address health needs. Another caretaker explained,

*‘I wasn’t sure how I felt about the clinic’s services, but I knew the vaccine was important, so I got it for my daughter. Sometimes, even if we have doubts about the system, we can’t let those doubts get in the way of what matters most.’ (FGD 7, Female 32 years)*

This highlights how caretakers are often willing to act decisively to protect their children’s health, regardless of uncertainties about healthcare services. Notably, the distance to health facilities did not significantly affect vaccination decisions. Caretakers living 2–5 km from the facility showed slightly lower odds of vaccinating (OR = 0.866, 95% CI = 0.111–6.743, *p* = 0.891), as did those 6–10 km away (OR = 0.358, 95% CI = 0.048–2.654, *p* = 0.315). However, those residing more than 10 km away had higher odds of vaccination (OR = 3.136, 95% CI = 0.521–18.881, *p* = 0.212), though these results did not reach statistical significance. One caretaker reflected,

*‘Yes, the health centre is far, but I am determined to try for my child. When it comes to their health, distance is not a concern.’ (FGD 8, Male 25 years)*

For many caretakers, the importance of vaccination surpasses the inconvenience of distance. Another participant added,

*‘I live in a very remote area, and walking that far can be exhausting, but I’ve learned that my child’s health comes first. If that means travelling a long way, so be it.’ (FGD 9, Female 57 years)*

It highlights the significant dedication many caretakers show to ensure their children’s vaccination despite experiencing logistical challenges. Similarly, transportation costs did not significantly impact vaccination decisions. Caretakers who spent < KSh. 100 on transport showed slightly reduced odds of vaccination (OR = 0.742, 95% CI = 0.100–5.489, *p* = 0.770) compared to those accessing the facility for free. Those spending KSh. 110–200 (OR = 0.515, 95% CI = 0.109–2.437, *p* = 0.402) or > KSh. 200 (OR = 0.885, 95% CI = 0.158–4.937, *p* = 0.889) also showed no significant differences. One caretaker shared,

*‘Yes, transport is expensive, but if my child were sick because I didn’t vaccinate them, that would be much worse. I find a way to make it work.’ (FGD 10, Female 28 years)*

This sentiment reveals that many caretakers prioritise the long-term benefits of vaccination over the short-term financial burdens of transportation. Another participant emphasised,

*‘The cost of getting to the clinic isn’t easy for me, but I’m willing to go into debt to have my daughter vaccinated. Health comes first.’ (FGD 11, Female 40 years)*

Despite financial constraints, caretakers often find ways to ensure their children receive the necessary vaccinations, illustrating a deep commitment to their children’s well-being. The findings of the binary regression model in this section have been summarised in Table 7 below.

## Discussion

The study findings indicate that age alone is not a consistent predictor of HPV vaccination decisions. Younger caretakers aged 26–34 were slightly less likely to vaccinate than those aged 18–25, while older caretakers aged 35–44 showed a moderate increase in likelihood. These trends align with a recent study suggesting younger caretakers often hesitate due to limited knowledge and resources, whereas older caretakers act more decisively, driven by greater awareness of HPV risks [18]. This highlights the need for universally accessible, targeted information to address knowledge gaps across all age groups.

Caretakers uncertain about HPV transmission through vaginal or anal routes were significantly more likely to vaccinate their children. This cautious behaviour supports findings by Liu *et al* [20] and Gallagher *et al* [19], which show that emphasising the vaccine’s protective benefits, even without a detailed understanding of transmission, can effectively motivate vaccination. Messaging that focuses on health benefits rather than complex details proves more impactful.

Unexpectedly, negative experiences with health workers sometimes encouraged proactive vaccination behaviours. While negative interactions often erode trust, intrinsic parental motivations can override such barriers [12, 14]. Nonetheless, fostering trust and positive relationships with healthcare providers remains essential for sustainable vaccination programs.

Satisfaction with healthcare services played a weaker, but notable, role. Caretakers expressing ambiguous satisfaction were more likely to vaccinate, prioritising health outcomes over service quality. Similar trends were noted by Kolek *et al* [18], emphasising that parental commitment to children’s health often transcends dissatisfaction with services.

Logistical challenges, such as distance to facilities and transport costs, did not significantly deter vaccination. Participants living over 10 km from a facility showed higher odds of vaccinating, though the results were not statistically significant. This mirrors findings in a systematic review, where caretakers prioritise their children’s health despite geographic and financial barriers [21]. These findings underscore the resilience and dedication of caretakers in ensuring their children’s well-being.

## Conclusion

Several factors influence HPV vaccination decisions among caretakers, including age, knowledge, healthcare experiences and logistical challenges. While older caretakers showed slightly higher vaccination rates, knowledge gaps were a universal concern, emphasising the need for targeted, accessible information.

Simplified messaging focusing on the vaccine’s protective benefits proved more effective than detailed explanations. Negative healthcare experiences occasionally motivated vaccination, though fostering trust with providers remains crucial.

Logistical challenges, such as distance and costs, had minimal impact, highlighting caretakers’ resilience and prioritising their children’s health. These findings underscore the importance of comprehensive, empathetic strategies to support informed vaccination decisions.

## Conflicts of interest

All authors affirm that no financial, personal or professional conflicts could have influenced the findings or interpretations. The research was conducted independently, and its conclusions are solely based on the data collected and analysed during the study.

## Funding

This study received no specific grant or funding from public, commercial or not-for-profit organisations. All research activities were conducted independently, with resources provided by the authors and their affiliated institutions.

## Institutional review

The study was reviewed and approved by the Moi University/Moi Teaching and Referral Hospital Ethics Committee (IREC-628/2023)

## Author contributions

Jared Otieno Ogolla conceptualised the study, collected and performed data analysis and drafted the manuscript. Dr David Rocaztle Masinde and Dr Alexander Munyao Mbeke provided oversight for the research process, offering critical guidance during the study design, methodology and data interpretation phases.

## Figures and Tables

**Table 1.** Socio-demographic characteristics of the study participants.

Characteristics of the study participant	*n* (%)
Age of the caretaker (in years) 18–25 26–34 35–44 45–54 ≥55	191 (11.8) 549 (33.8) 506 (31.2) 326 (20.0) 52 (3.2)
Age of their child (in years) 9–11 12–14	827 (50.9) 797 (49.1)
Education level None Primary Secondary Tertiary	865 (53.3) 435 (26.8) 81 (1.0) 243 (15.0)
Occupation None Informal employment Formal employment	387 (23.8) 821 (50.6) 416 (25.6)
Monthly income (in KSh) ≤10,000 11,000–20,000 21,000–30,000 31,000–40,000 41,000–50,000 ≥50,000	596 (36.7) 558 (34.4) 224 (13.8) 173 (10.7) 50 (3.1) 23 (1.3)
Type of house Permanent house Semi-permanent Mud/iron sheet walled	403 (24.8) 772 (47.6) 449 (27.6)
Total	1,624 (100)

**Table 2.** Relationship between socio-demographic characteristics of the caretakers and their daughters’ HPV vaccination status.

Characteristics of the caretakers	Pretest *n* (%)		*p*-value
	Vaccinated	Non-vaccinated	
Age of the caretaker (in years) 18–25 26–34 35–44 45–54 ≥55	18 (7.1) 111 (43.7) 73 (28.7) 46 (18.1) 6 (2.4)	43 (7.7) 181 (32.4) 214 (38.4) 105 (18.8) 15 (2.7)	*0.027
Age of their child (in years) 9–11 12–14	122 (48.0) 132 (52.0)	294 (52.7) 264 (47.3)	0.218
Education level None Primary Secondary Tertiary	139 (54.7) 63 (24.8) 16 (6.3) 36 (14.2)	287 (51.5) 157 (28.1) 29 (5.2) 85 (15.2)	0.662
Occupation None Informal employment Formal employment	63 (24.8) 116 (45.7) 75 (29.5)	134 (24.0) 290 (52.0) 134 (24.0)	0.174
Monthly income (in KSh) ≤10,000 11,000–20,000 21,000–30,000 31,000–40,000 41,000–50,000 ≥50,000	88 (34.6) 87 (34.3) 33 (13.0) 30 (11.8) 13 (5.1) 3 (1.2)	212 (38.0) 193 (34.6) 76 (13.6) 53 (9.5) 13 (2.3) 11 (2.0)	0.272
Type of house Permanent house Semi-permanent Mud/iron sheet walled	55 (21.7) 116 (45.7) 83 (32.6)	128 (22.9) 275 (49.3) 155 (27.8)	0.362

**Table 3.** Relationship between socio-cultural factors and the HPV vaccination status.

Socio-cultural factors	Vaccinated	Non-vaccinated	
The parent or guardian seeks permission before having the child vaccinated. Yes No I don’t know	83 (32.7) 122 (48.0) 49 (19.3)	206 (36.9) 244 (43.7) 108 (19.4)	0.449
The person whose permission is sought before having the child vaccinated Mother/Father-in-law Sister/Brother-in-law My parents Husband Sister Brother Uncle Aunt	12 (14.5) 14 (16.9) 8 (9.6) 18 (21.7) 13 (15.7) 12 (14.4) 3 (3.6) 3 (3.6)	14 (6.8) 34 (16.5) 26 (12.6) 44 (21.4) 36 (17.5) 30 (14.6) 12 (5.8) 10 (4.8)	0.637
All spouses are present during the child's vaccination. Yes No I don’t know	91 (35.8) 100 (39.4) 63 (24.8)	201 (36.0) 214 (38.4) 143 (25.6)	0.953
Reason for the spouse's absence during vaccination. It’s a taboo/untraditional Men are not allowed to go to a health clinic with women Busy at work He’s not interested I don’t know the reason My spouse believes it is my responsibility	57 (35.0) 43 (26.4) 25 (15.3) 20 (12.3) 15 (9.2) 3 (1.8)	135 (37.8) 88 (24.6) 51 (14.3) 47 (13.2) 24 (6.7) 12 (3.4)	0.799
Do cultural beliefs against vaccinating children exist? Yes No I don’t know	87 (34.2) 100 (39.4) 67 (26.4)	191 (34.2) 231 (41.4) 136 (24.4)	0.796
Cultural beliefs Religion Might cause infertility Might cause children not to grow well It makes young girls sexually active There is just something hidden about vaccines that we are not being told about The vaccine is not safe	14 (16.1) 27 (31.0) 12 (13.8) 17 (19.6) 12 (13.8) 5 (5.7)	30 (15.7) 41 (21.5) 33 (17.3) 40 (20.9) 39 (20.4) 8 (4.2)	0.481

**Table 4.** Relationship between caretakers’ knowledge levels and their daughters’ HPV vaccination status.

Caretakers’ knowledge	Pretest *n* (%)		
	Vaccinated	Non-Vaccinated	p-value
Having warts is a sign of HPV infection Yes No Don't know	94 (37.0) 92 (36.2) 68 (26.8)	177 (31.7) 190 (34.1) 191 (34.2)	0.093
One can contract HPV via anal/ vaginal intercourse Yes No Don't know	93 (36.6) 107 (42.1) 54 (21.3)	177 (31.7) 199 (35.7) 182 (32.6)	0.004
Vaginal bleeding after or during sex is a sign of HPV infection Yes No Don't know	101 (39.8) 106 (41.7) 47 (18.5)	212 (38.0) 253 (45.3) 93 (16.7)	0.607
Swelling/itching or discomfort at the location of the warts is a sign of HPV Yes No Don't know	98 (38.6) 105 (41.3) 51 (20.1)	193 (34.6) 234 (41.9) 131 (23.5)	0.429
Causes of HPV Multiple sexual partners Weak Immune system Personal contact	92 (36.2) 118 (46.5) 44 (17.3)	220 (39.4) 218 (39.1) 120 (21.5)	0.118
The HPV vaccine offers protection against most cervical cancers Yes No Don't know	87 (34.2) 114 (44.9) 53 (20.9)	193 (34.6) 254 (45.5) 119 (19.9)	0.950
Girls who have had the HPV vaccine do not need a Pap test when they are older Yes No Don't know	87 (34.3) 114 (44.9) 53 (20.9)	186 (33.3) 236 (42.3) 136 (24.4)	0.539
When is it recommended for the child to take the HPV vaccine? < 9 years 9–14 years >14 years	80 (31.5) 121 (47.6) 53 (20.9)	193 (34.6) 247 (44.3) 118 (21.1)	0.625
Is HPV vaccine free in public health facilities? Yes No Don't know	84 (33.1) 116 (45.7) 54 (21.2)	201 (36.0) 235 (42.1) 122 (21.9)	0.615
Is the HPV vaccine readily available in public health facilities? Yes No Don't know	90 (35.4) 114 (44.9) 50 (19.7)	183 (32.8) 239 (42.8) 136 (24.4)	0.332

**Table 5.** Relationship between the caretakers’ attitude towards HPV and HPV vaccine and their daughters’ HPV vaccination status.

The attitude of the caretaker towards HPV and HPV vaccine	Pretest *n* (%)		
	Vaccinated	Non-Vaccinated	*p*-value
The HPV vaccine is expensive Strongly agree Agree Neutral Disagree Strongly disagree	35 (13.8) 84 (33.1) 62 (24.4) 61 (24.0) 12 (4.7)	73 (13.1) 168 (30.1) 178 (31.9) 120 (21.5) 19 (3.4)	0.271
The HPV vaccine offers protection against cervical cancer Strongly agree Agree Neutral Disagree Strongly disagree	33 (13.0) 52 (20.5) 60 (23.6) 57 (22.4) 52 (20.5)	76 (13.6) 149 (26.7) 119 (21.3) 120 (21.5) 94 (16.8)	0.336
The HPV vaccine could cause fertility Strongly agree Agree Neutral Disagree Strongly disagree	22 (18.0) 37 (30.3) 29 (23.8) 21 (17.2) 13 (10.7)	55 (19.4) 70 (24.6) 76 (26.8) 57 (20.1) 26 (9.2)	0.739
The HPV vaccine could cause serious infections in children Strongly agree Agree Neutral Disagree Strongly disagree	65 (25.6) 65 (25.6) 59 (23.2) 41 (16.1) 24 (9.4)	125 (22.4) 144 (25.8) 109 (19.5) 124 (22.2) 56 (10.0)	0.272
It is good to vaccinate a child for HPV when they are 9–14 years old Strongly agree Agree Neutral Disagree Strongly disagree	46 (18.1) 90 (35.4) 55 (21.7) 46 (18.1) 17 (6.7)	105 (18.8) 182 (32.6) 128 (22.9) 106 (19.0) 37 (6.6)	0.956
The HPV vaccine is painful when administered to my child Strongly agree Agree Neutral Disagree Strongly disagree	53 (20.9) 67 (26.4) 63 (24.8) 48 (18.9) 23 (9.1)	128 (22.9) 161 (28.9) 113 (20.3) 107 (19.2) 49 (8.8)	0.661
It’s my responsibility to ensure my child receives HPV vaccine Strongly agree Agree Neutral Disagree Strongly disagree	38 (15.0) 56 (22.0) 48 (18.9) 59 (23.2) 53 (20.9)	68 (12.2) 129 (23.1) 125 (22.4) 116 (20.8) 120 (21.5)	0.620

**Table 6.** Relationship between health system factors and HPV vaccine and their daughters’ HPV vaccination status.

Health systems factors	Pretest *n* (%)		
	Vaccinated	Non-Vaccinated	p-value
Have you ever taken a child for any vaccination? Yes No	136 (53.5) 118 (46.5)	312 (55.9) 246 (44.1)	0.529
How long did it take before the child was vaccinated? (hours) < 1 1–-2 3–4 >4	64 (47.1) 45 (33.1) 7 (5.1) 20 (14.7)	164 (52.6) 83 (26.6) 17 (5.4) 48 (15.4)	0.572
What was the attitude of the health workers who served you? Good Average Poor Do not know	65 (47.8) 42 (30.9) 8 (5.9) 21 (15.4)	122 (39.1) 91 (29.2) 47 (15.1) 52 (16.6)	0.038
Were you satisfied with the services received at the facility? Yes No Do not know	63 (46.3) 61 (44.9) 12 (8.8)	145 (46.5) 111 (35.6) 56 (17.9)	0.026
How far was the health facility from your home? (km) <1 2–5 6–10 > 10	52 (38.2) 55 (40.4) 8 (5.9) 21 (15.4)	102 (32.7) 83 (26.6) 17 (5.4) 110 (35.3)	<0.001
Fare to the health facility Free, walking distance <100 110–200 >200	47 (34.6) 53 (39.0) 12 (8.8) 24 (17.6)	94 (30.1) 81 (26.0) 22 (7.1) 115 (36.8)	<0.001
Missed any vaccine at the facility? Yes No I don’t know	28 (20.6) 98 (72.1) 10 (7.3)	81 (26.0) 205 (65.7) 26 (8.3)	0.406
Reasons for missing the vaccine Lack of vaccine HPV vaccination health workers are not present Too many patients at the facility/overwhelmed health workers Long waiting time	8 (28.6) 9 (32.1) 2 (7.1) 9 (32.1)	28 (34.6) 19 (23.4) 9 (11.1) 25 (30.9)	0.759

**Table 7.** Binary regression table of factors associated with HPV vaccine uptake.

	B	S.E.	Wald	df	Sig.	OR	95% C.I. for OR	
							Lower	Upper
Age of the caretaker (in years)								
18–25			11.418	4	0.022			
26–34	−0.198	0.450	0.193	1	0.660	.821	.340	1.981
35–44	0.658	0.456	2.083	1	0.149	1.930	.790	4.716
45–54	0.379	0.482	0.619	1	0.431	1.461	.568	3.760
≥55	0.655	0.792	0.682	1	0.409	1.924	.407	9.094
One can contract HPV via anal/vaginal intercourse								
Yes			5.567	2	0.062			
No	0.124	0.253	0.242	1	0.623	1.132	0.690	1.858
Don’t know	0.705	0.308	5.245	1	0.022	2.024	1.107	3.701
What was the attitude of the health workers who served you?								
Good			14.232	3	0.003			
Average	0.533	0.325	2.683	1	0.101	1.704	.901	3.223
Poor	1.586	0.500	10.076	1	0.002	4.883	1.834	12.999
Do not know	−0.322	0.373	0.742	1	0.389	0.725	0.349	1.507
Were you satisfied with the services received at the facility?								
Yes			4.109	2	0.128			
No	−0.032	0.341	0.009	1	0.926	0.969	0.497	1.890
Don’t know	0.907	0.480	3.568	1	0.059	2.476	0.967	6.342
How far was the health facility from your home? (km)								
<1			3.295	3	0.348			
2–5	−0.144	1.047	0.019	1	0.891	.866	0.111	6.743
6–10	−1.027	1.022	1.010	1	0.315	.358	0.048	2.654
>10	1.143	0.916	1.557	1	0.212	3.136	0.521	18.881
Fare to the health facility (KSh)								
Free, walking distance			0.702	3	0.873			
<100	−0.299	1.021	0.086	1	0.770	0.742	0.100	5.489
110–200	−0.664	0.793	0.701	1	0.402	0.515	0.109	2.437
>200	−0.123	0.877	0.020	1	0.889	0.885	0.158	4.937
Constant	0.015	0.460	0.001	1	0.974	1.015		
